# Sigma-1 Receptor Inhibition Reduces Neuropathic Pain Induced by Partial Sciatic Nerve Transection in Mice by Opioid-Dependent and -Independent Mechanisms

**DOI:** 10.3389/fphar.2019.00613

**Published:** 2019-06-12

**Authors:** Inmaculada Bravo-Caparrós, Gloria Perazzoli, Sandra Yeste, Domagoj Cikes, José Manuel Baeyens, Enrique José Cobos, Francisco Rafael Nieto

**Affiliations:** ^1^Department of Pharmacology, School of Medicine, University of Granada, Granada, Spain; ^2^Institute of Neuroscience, Biomedical Research Center, University of Granada, Granada, Spain; ^3^Biosanitary Research Institute, University Hospital Complex of Granada, Granada, Spain; ^4^Department of Human Anatomy and Embryology, School of Medicine, University of Granada, Granada, Spain; ^5^Drug Discovery and Preclinical Development, Esteve, Barcelona, Spain; ^6^Institute of Molecular Biotechnology, Vienna, Austria; ^7^Teófilo Hernando Institute for Drug Discovery, Madrid, Spain

**Keywords:** neuropathic pain, spared nerve injury, sigma-1 receptors, S1RA, endogenous opioid system, mechanical allodynia, cold allodynia, heat hyperalgesia

## Abstract

Sigma-1 (σ_1_) receptor antagonists are promising tools for neuropathic pain treatment, but it is unknown whether σ_1_ receptor inhibition ameliorates the neuropathic signs induced by nerve transection, in which the pathophysiological mechanisms and response to drug treatment differ from other neuropathic pain models. In addition, σ_1_ antagonism ameliorates inflammatory pain through modulation of the endogenous opioid system, but it is unknown whether this occurs during neuropathic pain. We investigated the effect of σ_1_ inhibition on the painful hypersensitivity associated with the spared nerve injury (SNI) model in mice. Wild-type (WT) mice developed prominent cold (acetone test), mechanical (von Frey test), and heat hypersensitivity (Hargreaves test) after SNI. σ_1_ receptor knockout (ခσ_1_-KO) mice did not develop cold allodynia and showed significantly less mechanical allodynia, although they developed heat hyperalgesia after SNI. The systemic acute administration of the selective σ_1_ receptor antagonist S1RA attenuated all three types of SNI-induced hypersensitivity in WT mice. These ameliorative effects of S1RA were reversed by the administration of the σ_1_ agonist PRE-084, and were absent in σ_1_-KO mice, indicating the selectivity of S1RA-induced effects. The opioid antagonist naloxone and its peripherally restricted analog naloxone methiodide prevented S1RA-induced effects in mechanical and heat hypersensitivity, but not in cold allodynia, indicating that opioid-dependent and -independent mechanisms are involved in the effects of this σ_1_ antagonist. The repeated administration of S1RA twice a day during 10 days reduced SNI-induced cold, mechanical, and heat hypersensitivity without inducing analgesic tolerance during treatment. These effects were observed up to 12 h after the last administration, when S1RA was undetectable in plasma or brain, indicating long-lasting pharmacodynamic effects. These data suggest that σ_1_ antagonism may have therapeutic value for the treatment of neuropathic pain induced by the transection of peripheral nerves.

## Introduction

Neuropathic pain is a debilitating chronic pain condition resulting from a lesion or disease of the somatosensory system (Colloca et al., 2017). The prevalence of neuropathic pain in the general population has been estimated in the range of 6.9–10% (van Hecke et al., 2014), and it is expected to rise in the future (Colloca et al., 2017). Despite the enormous efforts devoted to both clinical and preclinical research, neuropathic pain treatment remains an unmet clinical need (Finnerup et al., 2015).

The sigma-1 (σ_1_) receptor is a unique ligand-operated chaperone expressed at high levels in several key pain control areas in both the peripheral and central nervous system, where it interacts with different receptors and ion channels to modulate them (Su et al., 2016; Sánchez-Fernández et al., 2017). The pharmacology of σ_1_ receptors has been deeply studied, and there are currently selective σ_1_ agonists (such as PRE-084) and antagonists (such as S1RA), to study σ_1_ receptor function (Sánchez-Fernández et al., 2017). Substantial evidence points to a prominent role for these receptors in neuropathic pain of diverse etiology (Merlos et al., 2017; Sánchez-Fernández et al., 2017), and shows that pain-like behaviors are attenuated in σ_1_-knockout (KO) mice (de la Puente et al., 2009; Nieto et al., 2012) and in wild-type (WT) animals treated with σ_1_ receptor antagonists (Roh et al., 2008; Nieto et al., 2012; Romero et al., 2012; Gris et al., 2016; Kang et al., 2016). The mechanisms involved in the antineuropathic effects of σ_1_ inhibition are only partially known and have been well studied in the central nervous system, specifically in the dorsal spinal cord, where these receptors control central sensitization (reviewed in Sánchez-Fernández et al., 2017).

There are differences between the pathophysiological mechanisms and responses to drug treatment for neuropathic pain induced by different types of injury to the peripheral nerves (Aley and Levine, 2002; Baron et al., 2010; Hershman et al., 2014; Finnerup et al., 2015). In particular, different neuroplastic changes (Casals-Díaz et al., 2009) and gene expression profiles (Griffin et al., 2007; Costigan et al., 2010) have been reported after denervation or constriction/ligation of the sciatic nerve. Surgical interventions inevitably results in nerve transection, and as a consequence, significant number of patients experience neuropathic pain (Borsook et al., 2013). However, all studies to date on the role of σ_1_ receptors in neuropathic pain after mechanical injury to peripheral nerves has focused on models of sciatic nerve constriction/ligation (Roh et al., 2008; de la Puente et al., 2009; Espinosa-Juárez et al., 2017a); thus the role of σ_1_ receptors in neuropathic pain induced by nerve transection has never been explored. Transection of the tibial and common peroneal branches of the sciatic nerve results in persistent neuropathic pain in rodents, manifested by marked hypersensitivity in the territory of the intact sural branch. Hence, this neuropathic pain model is termed the spared nerve injury (SNI) model (Decosterd and Woolf, 2000).

In view of these antecedents, the first goal of the present study was to test whether the inhibition of σ_1_ receptors alleviated the painful hypersensitivity associated with SNI-induced neuropathic pain. This was investigated by comparing SNI-induced neuropathic hypersensitivity in WT and σ_1_-KO mice, and by testing the effects, in animals with neuropathy, of the acute and repeated administration of the selective σ_1_ antagonist S1RA, which is currently under clinical development for the treatment of neuropathic pain (Abadias et al., 2013; Bruna et al., 2018).

Opioid receptors have been described as part of the interactome of σ_1_ receptors. This is relevant since σ_1_ receptors physically interact with opioid receptors restraining their functioning (reviewed in Sánchez-Fernández et al., 2017), so that σ_1_ receptor inhibition enhances analgesia induced by opioid drugs in nociceptive pain at both central (Mei and Pasternak, 2002) and peripheral sites (Sánchez-Fernández et al., 2013; Sánchez-Fernández et al., 2014; Prezzavento et al., 2017), and can increase the antihyperalgesic effects of endogenous opioid peptides (EOPs) produced naturally by immune cells that accumulate at the inflamed site to relieve inflammatory pain (Tejada et al., 2017). During neuropathic pain there is a prominent recruitment of immune cells harboring EOPs at both peripheral and central sites (reviewed in Ref. Tejada et al., 2018). However, whether σ_1_ receptors modulate endogenous opioid analgesia in neuropathic pain remains completely unknown. Therefore, the second goal of this study was to evaluate the possible contribution of the endogenous opioid system to the antineuropathic effects induced by S1RA in the mouse model of SNI.

## Methods

### Animals

Most experiments were performed in 8- to 11-week-old female WT CD-1 mice (Charles River, Barcelona, Spain) and σ_1_-KO CD-1 mice (Laboratorios Esteve, Barcelona, Spain). Some experiments were performed on male mice from the same strain and genotypes. Taking into account that male mice are much more aggressive to other mice than female animals (Edwards, 1968), and that stress such us that induced by fights with the alpha male can induce opioid analgesia (Miczek et al., 1982), we considered that this behavior of male mice might be a confounder in our experiments in the context on the modulation of endogenous opioid analgesia by σ_1_ receptors. Therefore, we performed most experiments in female mice. However, we also tested male mice in some key experiments (see the Results section) to explore a possible sexual dimorphism in σ_1_-mediated modulation of SNI-induced hypersensitivity. Female animals were tested at random times throughout the estrous cycle. Mice were housed in colony cages with free access to food and water prior to the experiments, and were kept in temperature- and light-controlled rooms (22 ± 2°C, and light–dark cycle of 12 h). The experiments were done during the light phase (from 9:00 a.m. to 3:00 p.m.). Animal care was in accordance with international standards (European Communities Council Directive 2010/63), and the protocol of the study was approved by the Research Ethics Committee of the University of Granada, Spain.

### Spared Nerve Injury

Mice were anesthetized with isoflurane (2%), and SNI surgery was performed as previously described (Bourquin et al., 2006). Briefly, an incision was made in the left thigh skin and was followed by an incision made directly through the biceps femoris muscle, exposing the sciatic nerve and its three terminal branches (the sural, common peroneal, and tibial nerves). The tibial and common peroneal branches of the sciatic nerve were ligated with a silk suture and transected distally, while the sural nerve was left intact. In sham-operated control mice, the sciatic nerve terminal branches were exposed but neither ligated nor transected. The day of SNI surgery is referred to as day 0. In some mice, SNI surgery induced hypoesthesia/anesthesia in the territory of the paw innervated by the sural nerve, instead of inducing sensory hypersensitivity. This was considered to be a consequence of a failed surgery and the mice were discontinued from further evaluations. These mice accounted for less than 1% of the mice tested.

### Drugs and Drug Administration

#### Acute Treatment Protocol

We used two selective σ_1_ receptor ligands: the σ_1_ antagonist S1RA (E-52862.HCl; 4-[2-[[5-methyl-1-(2-naphthalenyl)-1*H*-pyrazol-3-yl]oxy]ethyl] morpholine) (8–128 mg/kg; DC Chemicals, Shanghai, China), and the σ_1_ agonist PRE-084 (2-[4-morpholinethyl]1-phenylcyclohexanecarboxylate hydrochloride) (Tocris Cookson Ltd., Bristol, United Kingdom) (Cobos et al., 2008). In addition, we used the following opioid receptor ligands: the opioid agonist morphine hydrochloride (0.5–2 mg/kg; General Directorate of Pharmacy and Drugs, Spanish Ministry of Health), the opioid antagonist naloxone hydrochloride and its peripherally restricted analog naloxone methiodide (Sigma-Aldrich, Madrid, Spain). The doses of S1RA s.c. (subcutaneously) and morphine used to reverse mechanical, heat, and cold hypersensitivities were determined in the experiments shown in the “Results” section. The dose of PRE-084 used in the present study (32 mg/kg, s.c.) was selected based on our previous studies (Entrena et al., 2009; Montilla-García et al., 2018). The doses of naloxone (1 mg/kg, s.c.) and naloxone methiodide (2 mg/kg, s.c.) are those used in our previous studies (Sánchez-Fernández et al., 2014; Tejada et al., 2017). All drugs were dissolved to their final concentrations in sterile physiological saline just before administration, and were administered subcutaneously (s.c.) in the interscapular area in an injection volume of 5 mL/kg. The control animals received the same volume of the drug solvent (saline) s.c. All drugs were administered 7 days after surgery, when pain hypersensitivity was fully developed, and their effects were tested as explained in the Behavioral Assays section. When the effects of the association of two different drugs were evaluated, each injection was performed in a different area of the interscapular zone. In all cases, behavioral evaluations after drug administration were performed by an observer blinded to the treatment.

#### Repeated (10 days) Treatment Protocol

Treatment was given twice a day (every 12 h) *via* the intraperitoneal (i.p.) route with S1RA 25 mg/kg or vehicle, since it has been previously described that S1RA was efficacious using this administration protocol in a model of neuropathic pain induced by nerve ligation (Romero et al., 2012). Treatment started in the day of surgery (first injection 30 min before the injury) and was maintained for up to day 9 (i.e., 10 days of treatment). The effects of treatments were evaluated on days 7 (30 min after the administration of S1RA or saline), 10 (12 h after the last administration of S1RA or saline), 11, and 14 after nerve injury (36 and 108 h after the last administration of S1RA or saline, respectively) in each animal. Behavioral evaluations after repeated drug administration were performed by an observer blinded to the treatment.

### Behavioral Assays

#### Time Course Studies

To elucidate the time course of SNI-induced pain hypersensitivity in WT and σ_1_-KO mice, the behavioral responses were tested before surgery (baseline value). Then SNI surgery was performed and behavioral tests were carried out 3, 7, 14, and 21 days after SNI in each animal.

To investigate the acute effects of drugs on pain-related behaviors associated with SNI, presurgery baseline responses were evaluated, and then SNI surgery was performed. Seven days after the surgical procedure, when SNI-induced mechanical, heat, and cold hypersensitivities were fully developed, pretreatment measurements were made (time 0) and then the drugs or saline were injected s.c., and the response of the animal to the nociceptive test was measured again 30, 90, and 180 min after the injection.

In all cases, each mouse was evaluated in only one nociceptive test and received drug treatment or saline only once. All behavioral evaluations were recorded by an observer blinded to the genotype and treatment.

#### Procedure to Measure Mechanical Allodynia

Mechanical allodynia was assessed with von Frey filaments according to the up–down method (Chaplan et al., 1994), with slight modifications. On each day of evaluation the mice were habituated for 60 min in individual transparent plastic boxes (7 × 7 × 13 cm) placed on a wire mesh platforms. After the acclimation period, filaments were applied to the plantar ipsilateral hind paw in the sural nerve territory, pressed upward to cause a slight bend in the fiber, and left in place for 2–3 s. Calibrated von Frey monofilaments (Stoelting, Wood Dale, IL, USA) with bending forces that ranged from 0.02 to 1.4 g were applied using the up–down paradigm, starting with the 0.6 g filament and allowing 10 s between successive applications. The response to the filament was considered positive if immediate licking/biting, flinching, or rapid withdrawal of the stimulated paw was observed. In each consecutive test, if there was no response to the filament, a stronger stimulus was then selected; if there was a positive response, a weaker one was then used.

#### Procedure to Measure Cold Allodynia

Cold allodynia was tested by gently touching the plantar skin of the hind paw with an acetone drop, as previously described (Nieto et al., 2008). On each day of evaluation the mice were housed and habituated for 30 min in individual transparent plastic enclosures (7 × 7 × 13 cm) with a floor made of wire mesh. Acetone was applied three times to the ipsilateral hind paw at intervals of 30 s, and the duration of biting or licking of the hind paw was recorded with a stopwatch and reported as the cumulative time of biting/licking in all three measurements. A cutoff time of 10 s was used in each of the three trials, because animals rarely licked their hind paw for more than 10 s. During the presurgery baseline evaluation we discarded ≈ 5% of the mice tested due to an exaggerated atypical response to the acetone (>5 s of cumulative responses to acetone in the three measures).

#### Procedure to Measure Heat Hyperalgesia

To measure heat hyperalgesia we used the Hargreaves method (Tejada et al., 2014), with slight modifications as previously described (Hargreaves et al., 1988). Mice were habituated for 2 h in individual plastic chambers (9 × 9 × 22 cm) placed on a glass floor maintained at 30°C. After habituation, a beam of radiant heat was focused to the plantar surface of the ipsilateral hind paw with a plantar test apparatus (IITC, Los Angeles, CA, USA), until the mouse made a withdrawal response. Each mouse was tested three times, and the latencies were averaged for each animal. At least 60 s were allowed between consecutive measurements. A cutoff latency time of 20 s was used in each measurement to avoid lesions to the skin and unnecessary suffering.

### Determination of the Concentration of S1RA in Plasma and Brain Tissue

Animals were treated as described in the Repeated (10 Days) Treatment Protocol section, and the concentration of S1RA in plasma and brain tissue was measured 30 min and 12 h after the last i.p. administration. Briefly, a terminal blood sample was drawn from each mouse by cardiac puncture at the appropriate time after vehicle or drug administration. Blood samples were collected in heparinized tubes and centrifuged at 2,000 × g for 10 min to obtain plasma. Immediately after blood extraction, whole brains were removed. Plasma samples and brains were stored at −80°C until analysis. Each brain was weighted and homogenized in 4 mL Dulbecco’s phosphate buffered saline immediately before drug concentrations were determined. Protein was precipitated with acetonitrile, and samples were analyzed by high-performance liquid chromatography–triple quadrupole mass spectrometry (HPLC-MS/MS) according to a previously described procedure (Romero et al., 2012). The concentration of the compound in plasma or brain was determined by least-squares linear regression with a calibration curve.

### Data Analysis

For behavioral studies, statistical analysis was carried out with two-way repeated-measures analysis of variance (ANOVA). For the study of the S1RA levels determined by HPLC-MS/MS assay, statistical analysis was performed with two-way ANOVA. The Bonferroni *post hoc* test was performed in all cases. The differences between values were considered significant when the *p*-value was below 0.05. All data were analyzed with SigmaPlot 12.0 software (Systat Software Inc., San Jose, CA, USA).

## Results

### Comparison of Spared Nerve Injury-Induced Neuropathic Pain in σ_1_ Receptor Knockout and Wild-Type Mice

**Figure 1:**
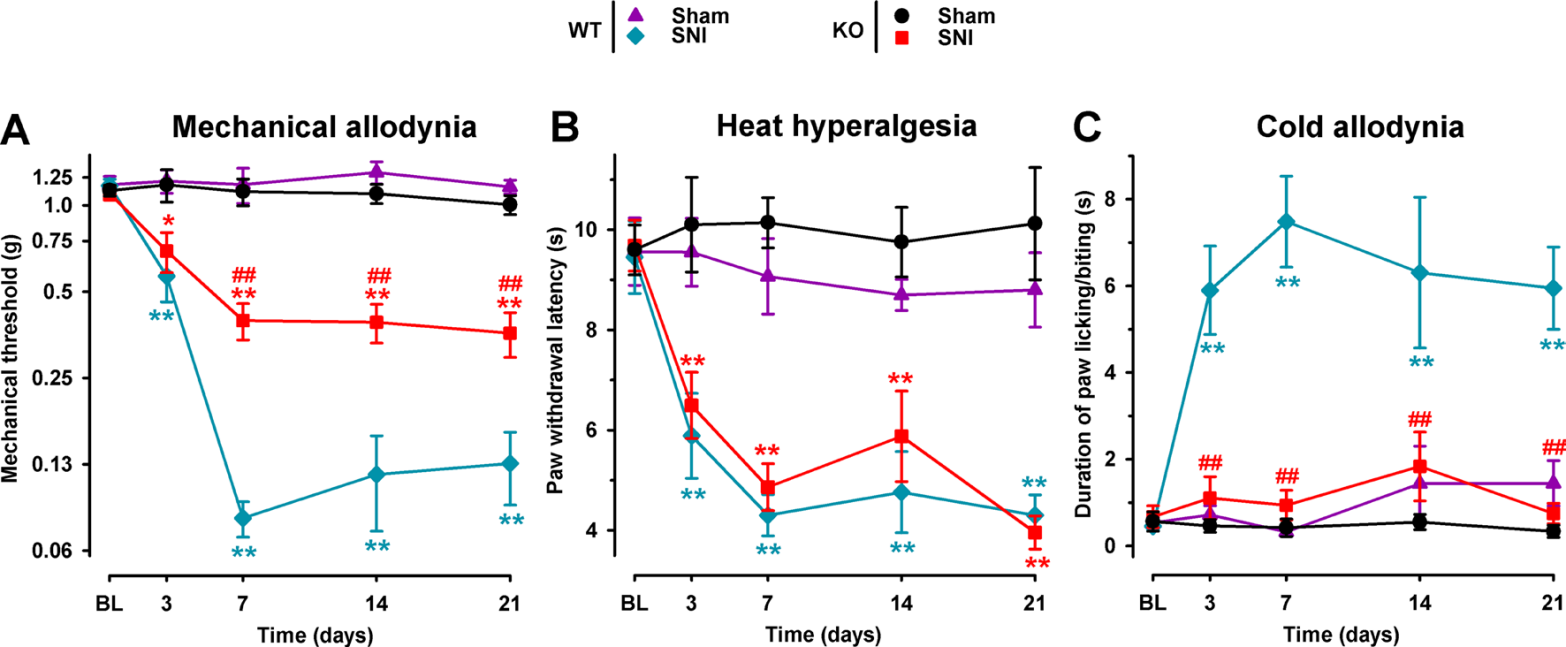
Comparison of spared nerve injury (SNI)-induced neuropathic pain behaviors in female wild-type (WT) and σ_1_ receptor knockout (KO) mice. The von Frey threshold **(A)**, latency to hind paw withdrawal in the Hargreaves test **(B)**, and duration of hind paw licking or biting in the acetone test **(C)** were recorded 1 day before (baseline, BL) and on days 3, 7, 14, and 21 after surgery in the paw ipsilateral to the surgery. Each point and vertical line represent the mean ± SEM of the values obtained in 10–12 animals. Statistically significant differences between the values in the sham and SNI groups on the same day: *P < 0.05; **P < 0.01; and among WT and KO groups: ^##^P < 0.01 (two-way repeated-measures ANOVA followed by Bonferroni test).

We studied the involvement of the σ_1_ receptor in neuropathic pain after SNI by comparing the response to mechanical, heat, and cold stimuli in WT and σ_1_-KO female mice. The baseline responses to the von Frey, Hargreaves, and acetone tests before surgery did not differ significantly between σ_1_-KO and WT animals in any group tested (**Figure 1A–C**). In the sham-operated groups there were no significant postsurgery changes in the responses to any of the three behavioral tests in either σ_1_-KO or WT animals (**Figure 1A–C**). However, after SNI surgery, WT mice developed mechanical allodynia, manifested as a significant reduction in the mechanical threshold, which was detectable as early as day 3 after surgery, was greatest on day 7, and remained observable throughout the 21-day evaluation period. σ_1_-KO mice also developed mechanical allodynia; however, it was significantly less pronounced than in WT mice, and the differences between WT and σ_1_-KO mice were statistically significant from day 7 to day 21 (**Figure 1A**). Both WT and σ_1_-KO mice developed a similar degree of heat hypersensitivity in the Hargreaves test after SNI, with paw withdrawal latencies to heat stimulation significantly lower than those in sham-operated mice of both genotypes and at all time points evaluated after SNI (**Figure 1B**). Wild-type mice with SNI also developed marked cold allodynia, manifested as a significantly longer increase in the duration of paw licking/biting induced by acetone from day 3 throughout the evaluation period in comparison to the control WT sham group (**Figure 1C**). In contrast, SNI surgery had no significant effect on the postsurgery responses in σ_1_-KO mice in the acetone test, given that the values in this group were virtually identical to those in the σ_1_-KO sham group throughout the evaluation period (**Figure 1C**). These results are summarized in **Table 1**.

**Table 1:** Summary of the main results obtained in this study on the effect of the acute administration of S1RA to female wild-type mice and the effects seen on of σ_1_ receptor knockout female mice on the spared nerve injury model of neuropathic pain. The figures that show the results for the different experiments are indicated.

Type of sensory hypersensitivity	Wild-type mice									σ_1_ receptor knockout mice			
	Efficacy*	Potency*	PRE-084			Naloxone*		Naloxone methiodide*		Without any treatment*		S1RA effects	
Mechanical allodynia	++	+	**Figure 3A**	Yes	**Figure 4A**	Yes	**Figure 6A**	Yes	**Figure 6A**	Partially reduced	**Figure 1A**	Absent	**Figure 5A**
Heat hyperalgesia	+++	++	**Figure 3B**	Yes	**Figure 4B**	Yes	**Figure 6B**	Yes	**Figure 6B**	Fully present	**Figure 1B**	Absent	**Figure 5B**
Cold allodynia	+++	+++	**Figure 3C**	Yes	**Figure 4C**	No	**Figure 6C**	Not tested		Absent	**Figure 1C**	Not tested	

**Figure 2:**
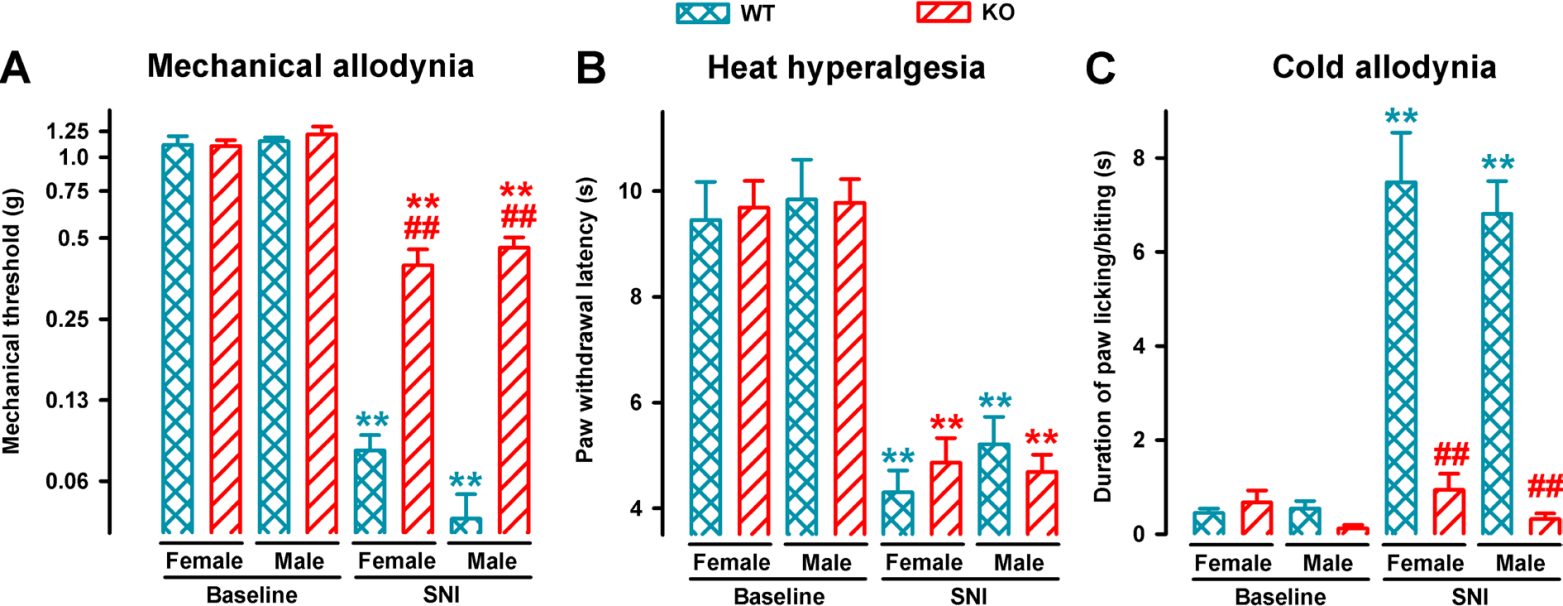
Comparison of SNI-induced neuropathic pain behaviors in female and male WT and σ_1_-KO mice. The von Frey threshold **(A)**, latency to hind paw withdrawal in the Hargreaves test **(B)**, and duration of hind paw licking or biting in the acetone test **(C)** were recorded 1 day before (baseline) and 7 days after surgery in the paw ipsilateral to the surgery. Each point and vertical line represent the mean ± SEM of the values obtained in 8–12 animals. Statistically significant differences between the values on the presurgery (baseline) day and 7 days after SNI in mice of the same sex: **P < 0.01; and between WT and KO groups of mice of the same sex: ^##^ *P* < 0.01. There were no statistical differences between values from mice of different sexes under the same experimental conditions (two-way repeated-measures ANOVA followed by Bonferroni test).

*Experiments where male mice were also tested with similar results to female mice (see **Figures 2** and **6**).

We also compared mechanical, heat, and cold hypersensitivities induced by SNI in female and male animals from both genotypes. On day 7 after SNI, sensory hypersensitivity to the three types of stimuli was equivalent in WT female and male mice (**Figure 2**). σ_1_-KO mice of both sexes showed an equivalent reduction of mechanical allodynia, while showing the same extent of heat hyperalgesia than WT mice, but no cold hypersensitivity (**Figure 2A–C**, respectively).

In summary, SNI surgery induced mechanical, heat, and cold hypersensitivity in WT mice of both sexes. However, SNI surgery led to a clearly different pattern of neuropathic signs in σ_1_-KO mice irrespectively of the sex tested, as these female or male mutant mice developed heat hyperalgesia, but did not develop cold allodynia and showed significantly less mechanical allodynia.

### Effects of the Acute Systemic Administration of the Selective σ_1_ Receptor Antagonist S1RA on Spared Nerve Injury-Induced Mechanical, Cold, and Heat Hypersensitivity

**Figure 3:**
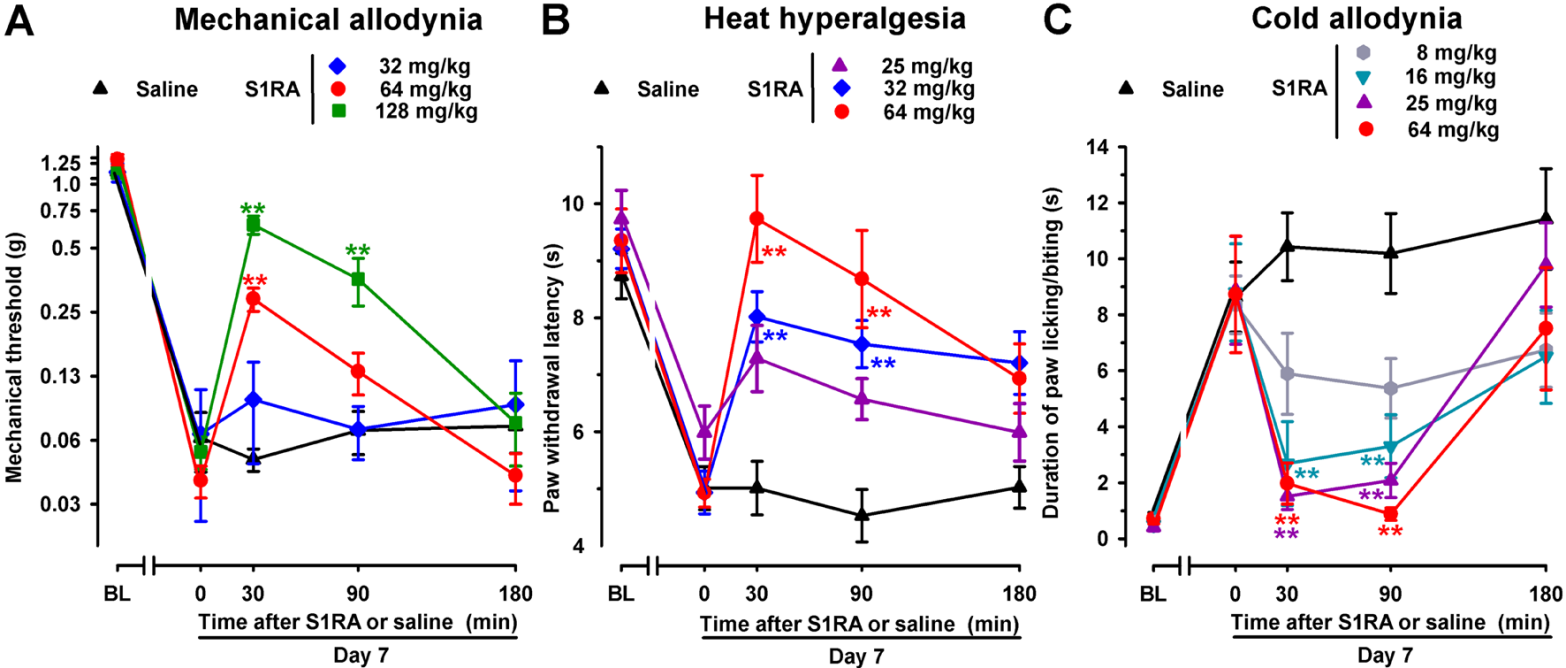
Time course of the effects of a single subcutaneous (s.c.) injection of S1RA (8–128 mg/kg) or saline on mechanical allodynia **(A)**, heat hyperalgesia **(B)**, and cold allodynia **(C)**, in female WT mice with SNI, 7 days after surgery. The von Frey threshold **(A)**, the latency to hind paw withdrawal in the Hargreaves test **(B)**, and the duration of hind paw licking or biting in the acetone test **(C)** were recorded 1 day before (baseline, BL) and 7 days (Day 7) after surgery in the paw ipsilateral to the surgery. On day 7, the responses to test stimuli was recorded immediately before (time 0) and at several times (30, 90, and 180 min) after injection of the drug or saline. Each point and vertical line represent the mean ± SEM of the values obtained in 8–11 animals. Statistically significant differences between S1RA- and saline-treated groups at the same time after treatment: **P < 0.01 (two-way repeated-measures ANOVA followed by Bonferroni test).

To test the effects of acute pharmacological antagonism by the σ_1_ receptor on SNI-induced neuropathic pain, the selective σ_1_ receptor antagonist S1RA was administered s.c. to female WT mice after neuropathy was fully developed (7 days after surgery). The threshold force needed to evoke pain-like responses before treatment with S1RA or saline was significantly lower than in the baseline measurement (**Figure 3A**, time 0), thus showing mechanical allodynia. Saline administration did not significantly modify SNI-induced mechanical allodynia during the 3-h test period (**Figure 3A**). In contrast, the acute administration of S1RA (32–128 mg/kg) attenuated mechanical allodynia in a dose-dependent manner (**Figure 3A**). In mice with SNI, paw withdrawal latencies to radiant heat were significantly shorter, in comparison to their baseline measurements, in all groups of animals before S1RA or saline administration (**Figure 3B**, time 0). Saline administration did not significantly modify SNI-induced heat hyperalgesia, whereas the s.c. administration of S1RA (25–64 mg/kg) dose-dependently inhibited this response (**Figure 3B**). In the acetone test, WT mice 7 days after SNI and before treatment with S1RA or saline (time 0) showed a longer duration of paw licking/biting in response to acetone (**Figure 3C**, time 0). In these mice, a single s.c. injection of saline did not modify the response to acetone at any of the time points tested (**Figure 3C**). However, a single s.c. injection of S1RA (8–64 mg/kg) dose-dependently reduced the duration of acetone-induced paw licking/biting from 30 to 90 min after treatment (**Figure 3C**). Among the three sensory modalities explored in female mice, cold allodynia was the most sensitive outcome to the effects of S1RA, as it was fully reversed by 16 mg/kg of this compound, whereas S1RA 64 mg/kg was needed to fully reverse heat hyperalgesia, and we had to increase the dose of S1RA up to 128 mg/kg to induce a prominent (although partial) effect on SNI-induced mechanical allodynia (compare **Figure 3A–C**). These results are summarized in **Table 1**.

**Figure 4:**
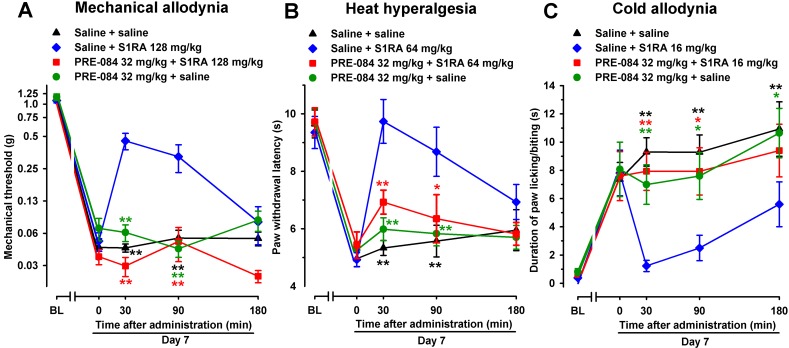
The σ_1_ receptor agonist PRE-084 reversed the effects of the σ_1_ receptor antagonist S1RA in female WT mice with SNI 7 days after surgery. Mechanical allodynia **(A)**, heat hyperalgesia **(B)**, and cold allodynia **(C)** were evaluated 1 day before (baseline, BL) and 7 days (day 7) after surgery, in the paw ipsilateral to the surgery. On day 7, the responses to test stimuli were recorded immediately before (time 0) and at several times (30, 90, and 180 min) after injection of the drugs or saline. Each point and vertical line represent the mean ± SEM of the values obtained in 8–14 animals. Statistically significant differences in comparison to the saline+S1RA group: *P < 0.05; **P < 0.01 (two-way repeated-measures ANOVA followed by Bonferroni test).

In contrast to the effect of the σ_1_ receptor antagonist S1RA, the selective σ_1_ agonist PRE-084 (32 mg/kg, s.c.), when tested 7 days after SNI, did not alter SNI-induced mechanical-, heat-, or cold-hypersensitivity in female WT mice (**Figure 4A–C**, respectively). However, when PRE-084 (32 mg/kg, s.c.) and S1RA were associated, the antiallodynic and antihyperalgesic effects of this σ_1_ antagonist were abolished (**Figure 4A–C**), suggesting that the effects of S1RA were mediated by the pharmacological antagonism of σ_1_ receptors.

**Figure 5:**
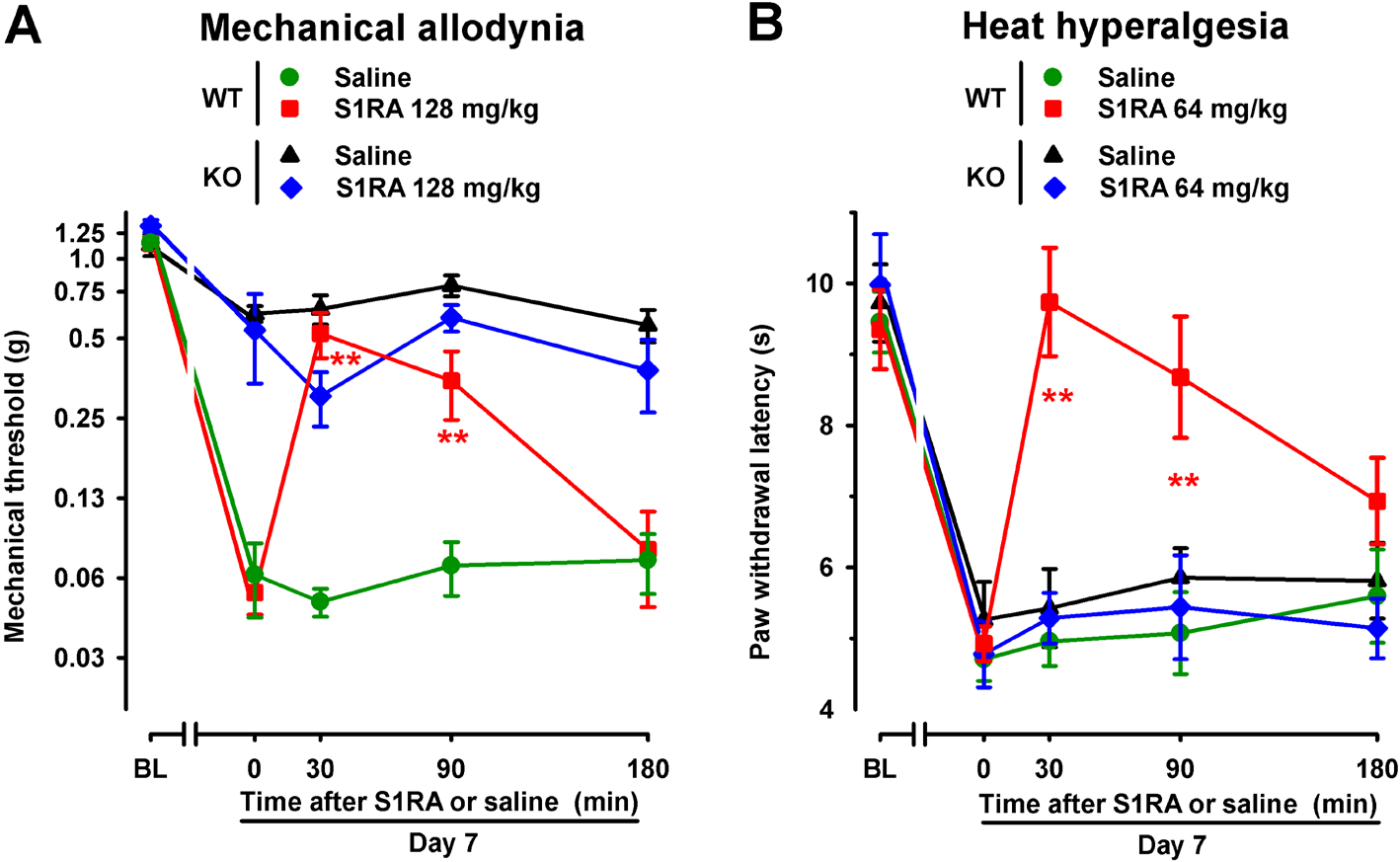
Time course of the effects of a single s.c. injection of S1RA (64–128 mg/kg) or saline on mechanical allodynia **(A)** and heat hyperalgesia **(B)** in female WT and σ_1_-KO mice with SNI 7 days after surgery. The von Frey threshold **(A)** and latency to hind paw withdrawal in the Hargreaves test **(B)** were evaluated 1 day before (baseline, BL) and 7 days (day 7) after surgery in the paw ipsilateral to the surgery. On day 7 the responses to test stimuli were recorded immediately before (time 0) and at several times (30, 90, and 180 min) after injection of the drug or saline. Each point and vertical line represent the mean ± SEM of the values obtained in 8–14 animals. Statistically significant differences between S1RA- and saline-treated groups at the same time after treatment were found in WT mice (**P < 0.01) but not in σ_1_-KO mice (two-way repeated-measures ANOVA followed by Bonferroni test).

To further verify the role σ_1_ receptors on the effects induced by S1RA in SNI-induced hypersensitivity, we compared its effects in female WT and mice lacking σ_1_ receptors (σ_1_-KO mice). We tested the effects of this drug in σ_1_-KO mice only for SNI-induced mechanical allodynia, which was partially developed in these mice, and heat hypersensitivity, which fully developed in σ_1_-KO mice; whereas we did not test for the possible effects of S1RA on cold allodynia, since this type of hypersensitivity was absent in σ_1_-KO mice (as shown in **Figure 1**). WT mice given the σ_1_ antagonist S1RA showed less SNI-induced mechanical allodynia (**Figure 5A**) and heat hyperalgesia (**Figure 5B**), but the administration of S1RA to σ_1_-KO mice did not induce significant antiallodynic or antihyperalgesic effects in these mutant mice (**Figure 5A and B**).

Therefore, both the reversion of the effects of S1RA by PRE-084 and the absence of activity of S1RA in mice lacking σ_1_ receptors suggest that off-target effects did not contribute to the antineuropathic effects of the σ_1_ antagonist S1RA in this neuropathic pain model.

### Contribution of the Endogenous Opioid System to Antineuropathic Effects of the Systemic Administration of S1RA on Spared Nerve Injury-Induced Neuropathic Pain

**Figure 6:**
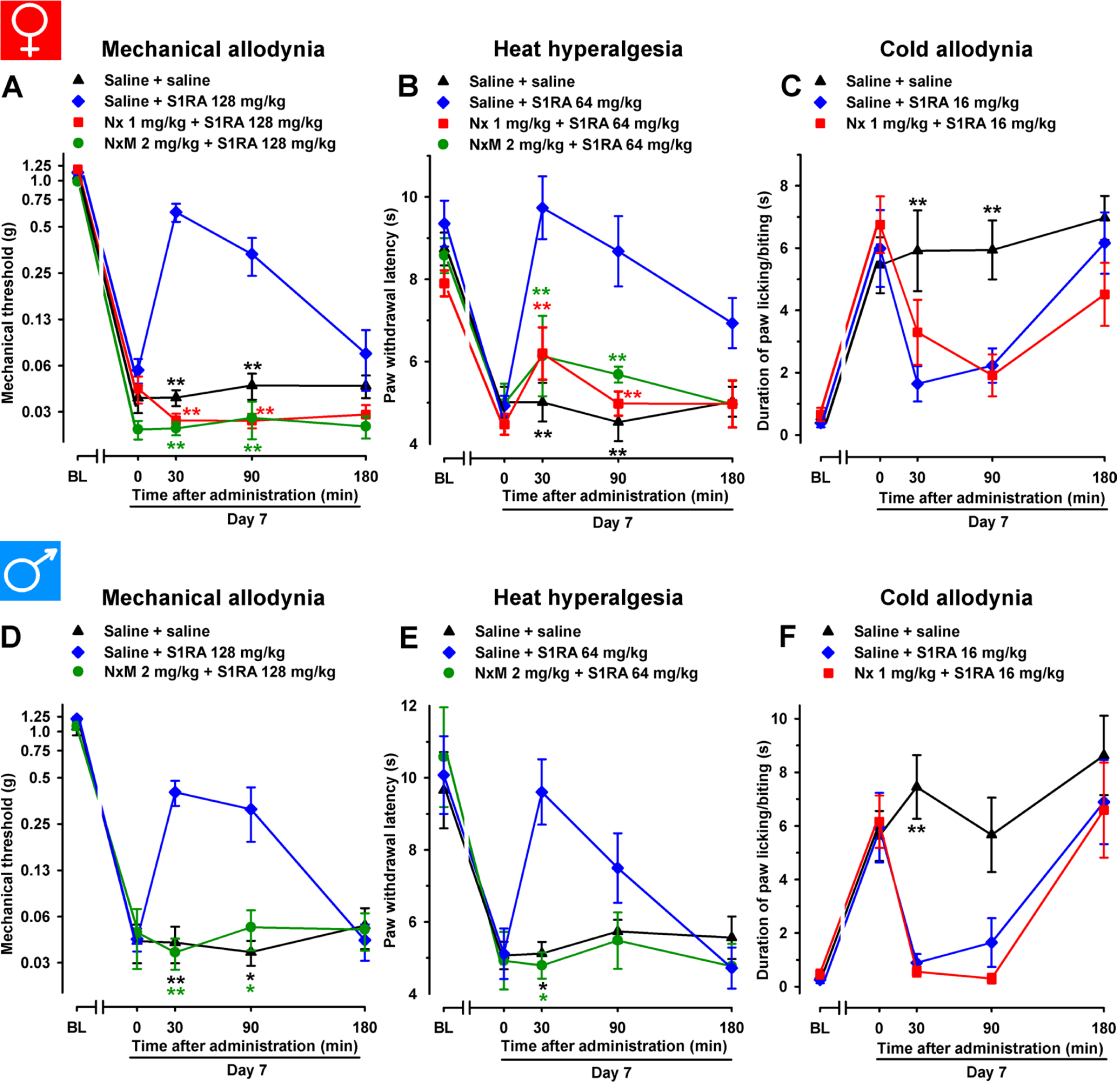
The opioid antagonists naloxone hydrochloride (Nx) and naloxone methiodide (NxM) reversed the effects of S1RA on mechanical allodynia and heat hyperalgesia but not on cold allodynia in female and male WT mice with SNI 7 days after surgery. In female mice, mechanical allodynia **(A)**, heat hyperalgesia **(B)**, and cold allodynia **(C)** were evaluated 1 day before (baseline, BL) and 7 days (day 7) after surgery. Identical procedures were performed on male mice for the determination of mechanical **(D)**, heat **(E)**, and cold **(F)** sensitivity. On day 7 the responses to test stimuli in the paw ipsilateral to the surgery were recorded immediately before (time 0) and at several times (30, 90, and 180 min) after injections of the drugs (S1RA and opioid antagonists) or saline. Each point and vertical line represent the mean ± SEM of the values obtained in 8–14 animals. Statistically significant differences in comparison to the saline+S1RA group: **P < 0.01 (two-way repeated-measures ANOVA followed by Bonferroni test).

In female WT mice, the association of the opioid antagonist naloxone (1 mg/kg, s.c.) with S1RA administered 7 days after SNI surgery completely reversed the ameliorative effects produced by the σ_1_ antagonist on hypersensitivity to mechanical (**Figure 6A**) and heat stimuli (**Figure 6B**). We also tested the effects of the peripherally restricted opioid antagonist naloxone methiodide on the antineuropathic effects of S1RA, and observed that peripheral opioid antagonism was also able to fully reverse the effects of S1RA on mechanical and heat hypersensitivity (**Figure 6A and B**). These data suggest that the effects induced by the σ_1_ antagonist on SNI-induced mechanical and heat hypersensitivity require the participation of the opioid system at the peripheral level. In contrast, naloxone treatment did not alter the effects of S1RA on cold allodynia (**Figure 6C**), suggesting that opioid-independent effects induced by S1RA were involved in the decrease in this type of hypersensitivity. These results are summarized in **Table 1**.

We tested these same doses of S1RA in male mice and found equivalent results than those found in female mice: the acute administration of the σ_1_ receptor antagonist S1RA to male mice partially reversed SNI-induced mechanical allodynia but completely reversed heat and cold hypersensitivity (**Figure 6D–F**, respectively). Similar to the results shown with female mice, the ameliorative effects of S1RA on mechanical and heat SNI-induced hypersensitivity in male mice were reversed by naloxone methiodide, whereas naloxone did not reverse the effects of S1RA on cold allodynia (**Figure 6D–F**, respectively).

**Figure 7:**
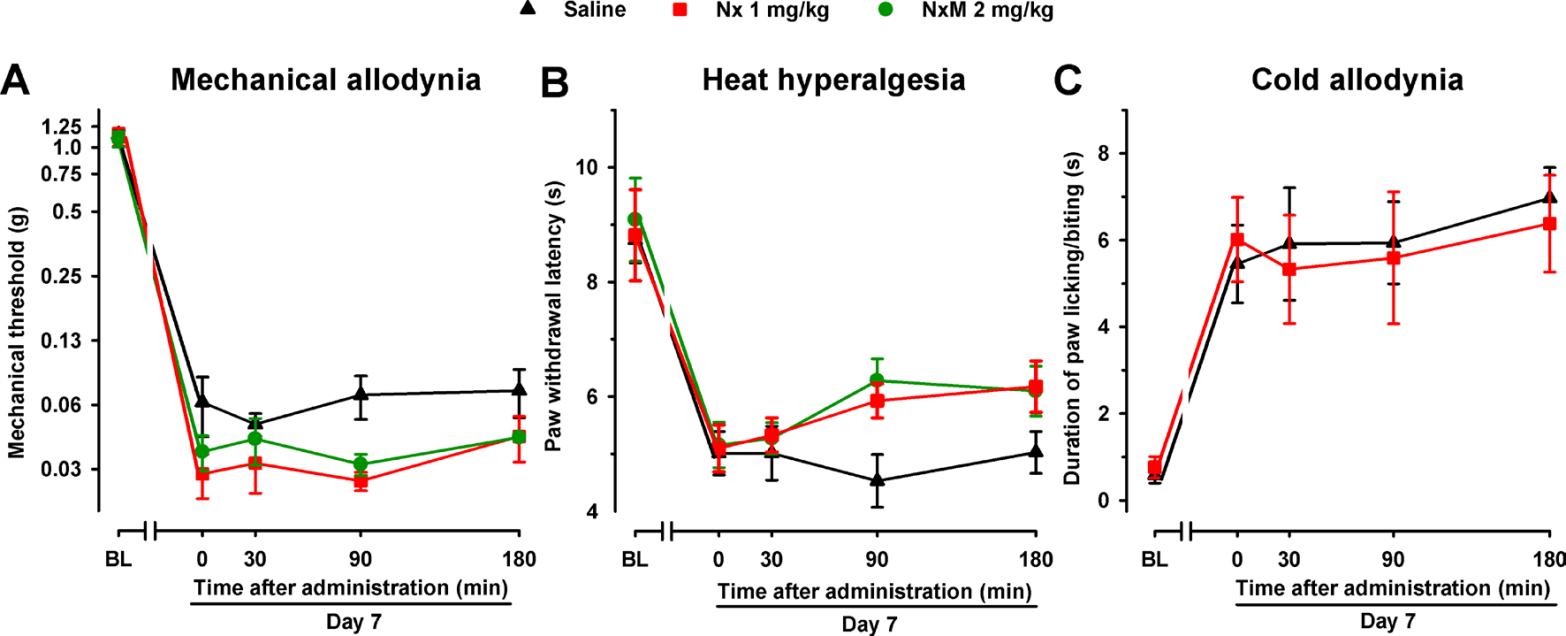
The opioid antagonists Nx and NxM had no effect per se in female WT mice with SNI 7 days after surgery. Mechanical allodynia **(A)**, heat hyperalgesia **(B)**, and cold allodynia **(C)** were evaluated 1 day before (baseline, BL) and 7 days (day 7) after surgery in the paw ipsilateral to the surgery. On day 7 the responses to test stimuli were recorded immediately before (time 0) and at several times (30, 90, and 180 min) after injection of the opioid antagonist or saline. Each point and vertical line represent the mean ± SEM of the values obtained in 8–10 animals. Neither of the treatments produced statistically significant differences in comparison to the saline group (two-way repeated-measures ANOVA followed by Bonferroni test).

In order to explore whether the endogenous opioid system influences pain hypersensitivity induced by SNI in female mice, we administered naloxone (1 mg/kg) and naloxone methiodide (2 mg/kg) in the absence of S1RA to WT mice 7 days after SNI. No significant effects were observed with any of the opioid antagonists in any of the three sensory modalities explored (**Figure 7A–C**).

### Effects of the Systemic Administration of Morphine on Spared Nerve Injury-Induced Cold and Mechanical Allodynia and Heat Hyperalgesia

**Figure 8:**
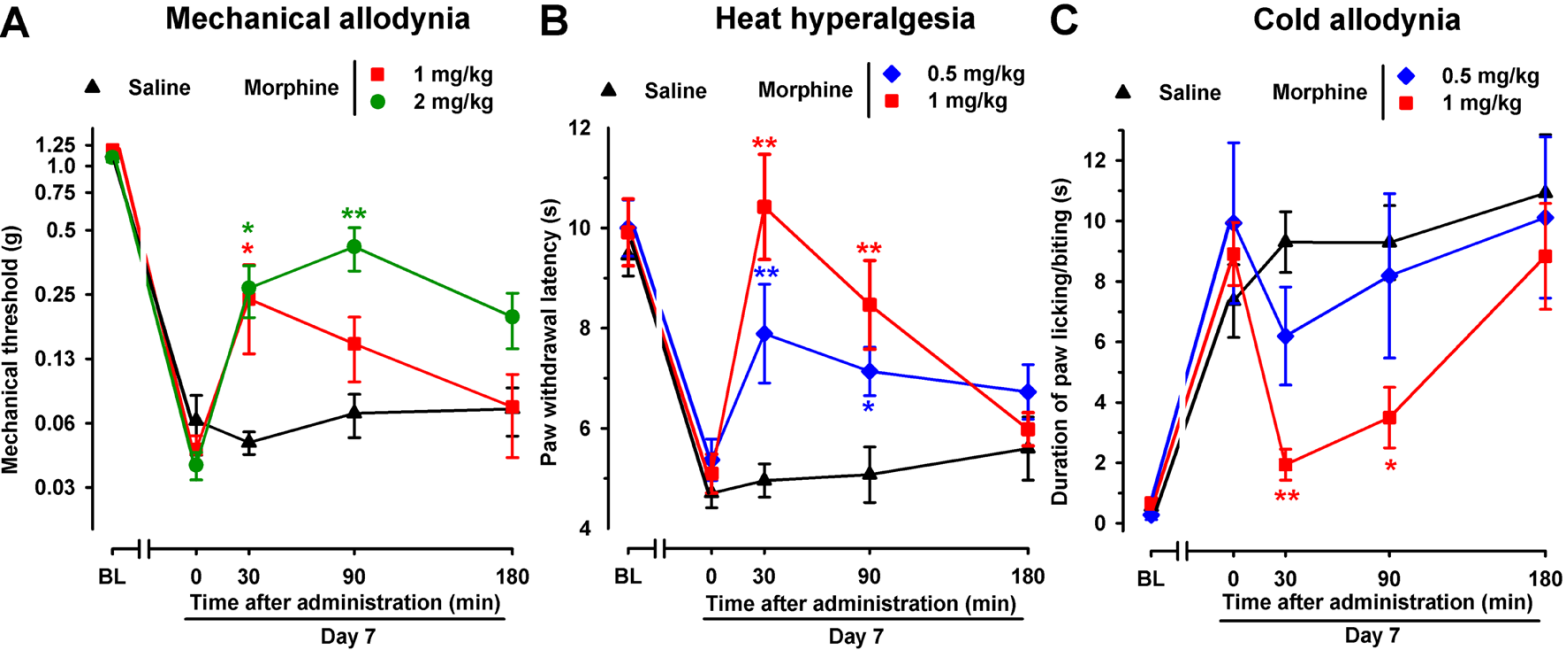
Time course of the effects of a single s.c. injection of morphine (0.5–2 mg/kg) or saline on mechanical allodynia **(A)**, heat hyperalgesia **(B)**, and cold allodynia **(C)** in female WT mice with SNI 7 days after surgery. The von Frey threshold (A), latency to hind paw withdrawal in the Hargreaves test **(B)**, and duration of hind paw licking or biting in the acetone test **(C)** were recorded 1 day before (baseline, BL) and 7 days (day 7) after surgery in the paw ipsilateral to the surgery. On day 7 the responses to test stimuli were recorded immediately before (time 0) and at several times (30, 90, and 180 min) after injection of the drug or saline. Each point and vertical line represent the mean ± SEM of the values obtained in 8–13 animals. Statistically significant differences between morphine- and saline-treated groups at the same time after treatment: *P < 0.05; **P < 0.01 (two-way repeated-measures ANOVA followed by Bonferroni test).

To test the effects of an opioid drug on SNI-induced sensory hypersensitivity, we evaluated the effects of morphine in this neuropathic pain model in female mice. As expected, acute administration of the morphine solvent (saline) had no effect on hypersensitivity following SNI surgery (**Figure 8A–C**). However, the administration of morphine (1 and 2 mg/kg, s.c.) led to significantly less mechanical allodynia associated with SNI, with a more prolonged effect at the highest dose tested (**Figure 8A**). In addition, acute treatment with morphine (0.5 and 1 mg/kg) inhibited, in a dose-dependent manner, both heat hyperalgesia (**Figure 8B**) and cold allodynia (**Figure 8C**) induced by SNI. Whereas a single s.c. injection of morphine (1 mg/kg) completely reversed heat and cold hypersensitivity induced by SNI, the effect of morphine on SNI-induced mechanical allodynia was weaker, leading to only a partial reduction (see **Figure 8A–C**).

**Figure 9:**
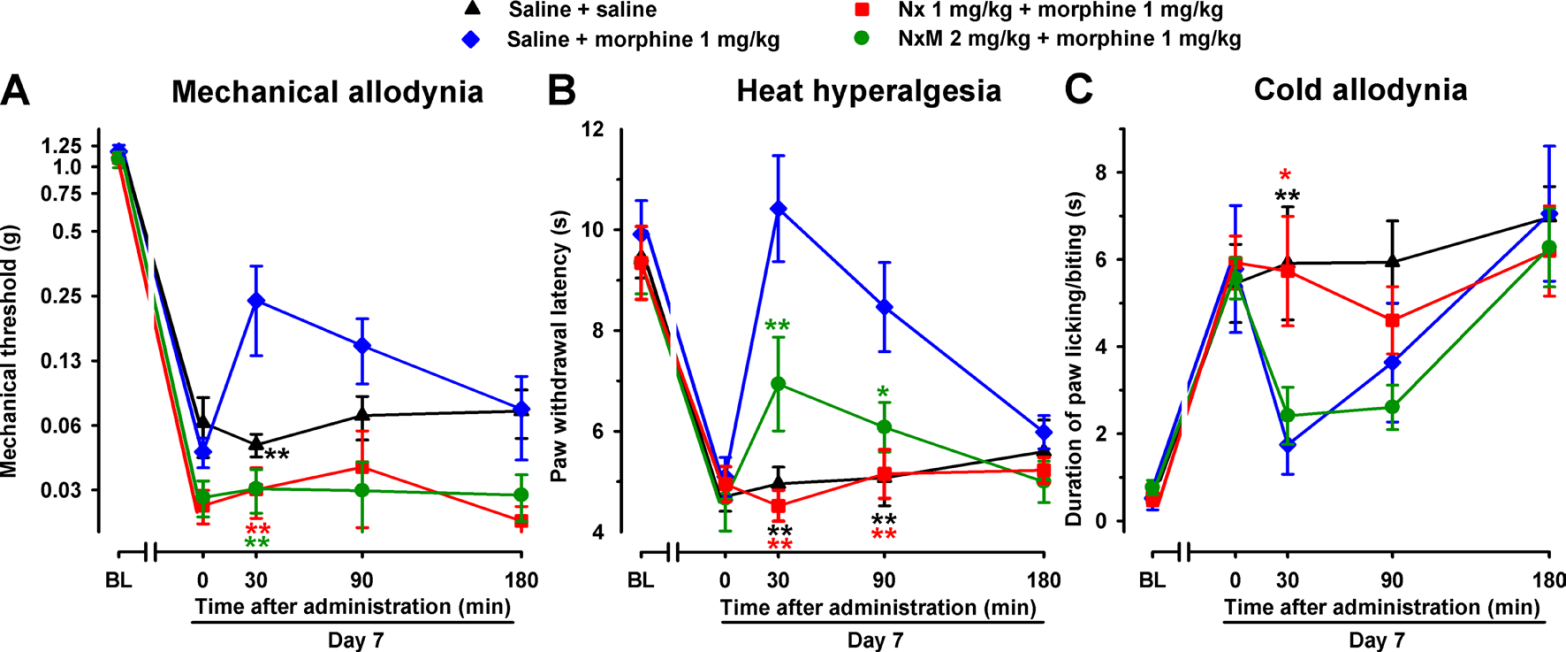
Differential ability of Nx and NxM to reverse the effects of morphine on mechanical allodynia, heat hyperalgesia, and cold allodynia in female WT mice with SNI 7 days after surgery. Mechanical allodynia **(A)**, heat hyperalgesia **(B)**, and cold allodynia **(C)** were evaluated 1 day before (baseline, BL) and 7 days (day 7) after surgery in the paw ipsilateral to the surgery. On day 7 the responses to test stimuli were recorded immediately before (time 0) and at several times (30, 90, and 180 min) after injections of the drugs (morphine and opioid antagonists) or saline. Each point and vertical line represent the mean ± SEM of the values obtained in 8–14 animals. Statistically significant differences in comparison to the saline+morphine group: *P < 0.05; **P < 0.01 (two-way repeated-measures ANOVA followed by Bonferroni test).

To elucidate the possible contribution of peripheral opioid receptors to the antinociceptive effects induced by morphine in SNI mice, we associated morphine administration with the injection of the opioid antagonists naloxone or naloxone methiodide. As expected, naloxone (1 mg/kg s.c.) completely reversed the antinociceptive effect of morphine in all three sensory modalities explored, with values indistinguishable from those in mice treated with the drug solvent (**Figure 9A–C**). However, naloxone methiodide (2 mg/kg) completely reversed the effect of morphine (1 mg/kg) on mechanical allodynia (**Figure 9A**), and markedly reduced its effects on heat hyperalgesia (**Figure 9B**), whereas it did not reverse the effect of morphine on SNI-induced cold allodynia (**Figure 9C**). These data suggest that peripheral opioid receptors contributed to the ameliorative effects induced by morphine only in hypersensitivity to mechanical and heat stimuli induced by SNI, but not in cold allodynia.

### Effect of Repeated Treatment With S1RA on Neuropathic Pain-Related Behaviors

**Figure 10:**
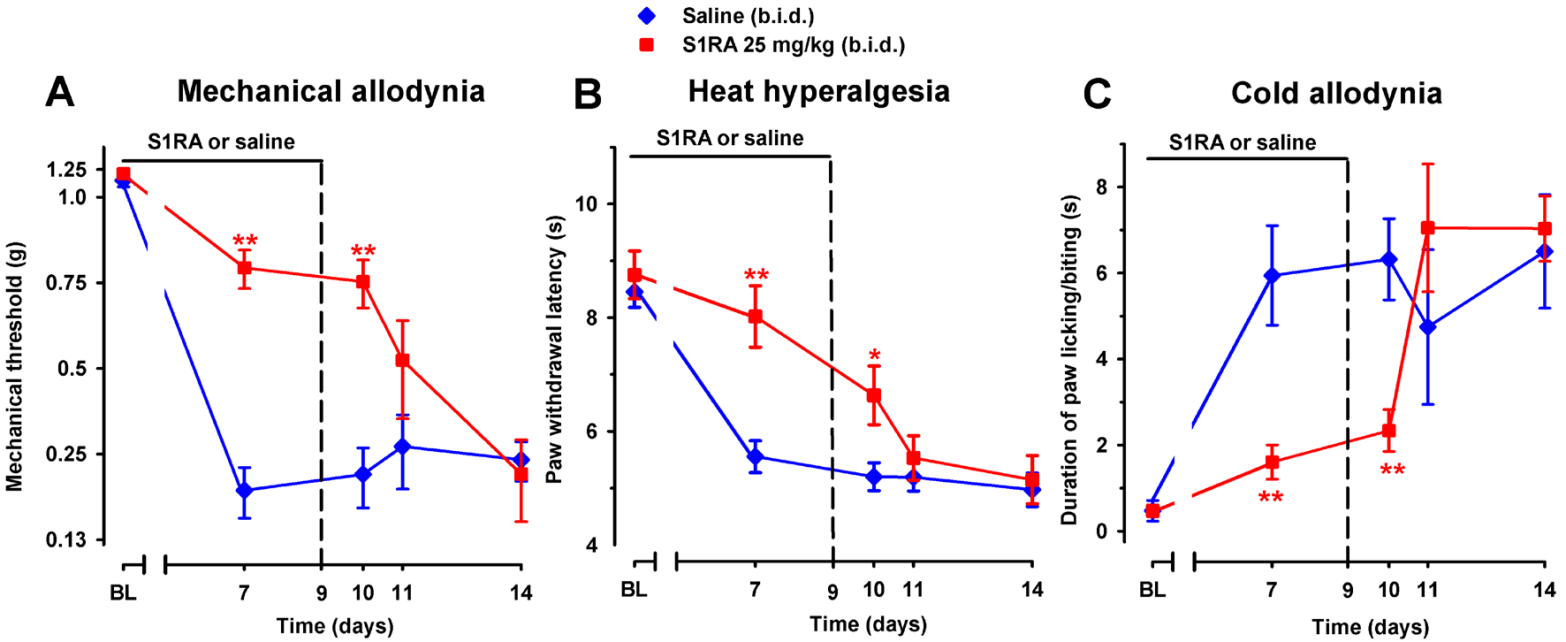
Time course of the effect of repeated treatment with S1RA on mechanical allodynia **(A)**, heat hyperalgesia **(B)**, and cold allodynia **(C)** induced by SNI in female WT mice. The mice were treated twice daily with either saline or S1RA (25 mg/kg, i.p.). The first injection was administrated 30 min before SNI surgery. Responses were recorded in each animal before SNI (baseline, BL) and 30 min after the administration of S1RA or saline on day 7 in the paw ipsilateral to the surgery. Treatment was continued until day 9 and the mice were evaluated again on days 10, 11, and 14 postsurgery. The black horizontal line at the top of each figure illustrates the duration of treatment with S1RA or saline. Each point and vertical line represent the mean ± SEM of the values obtained in 10–14 animals. Statistically significant differences between S1RA- and saline-treated groups on the same day after treatment: *P < 0.05; **P < 0.01 (two-way repeated-measures ANOVA followed by Bonferroni test).

To study the effect of prolonged pharmacological antagonism of the σ_1_ receptors on the development of SNI-induced neuropathy, we administered to WT female mice two daily injections of S1RA (25 mg/kg, i.p.) or saline, starting 30 min before surgery and continuing up to day 9. Mechanical allodynia (**Figure 10A**), heat hyperalgesia (**Figure 10B**), and cold allodynia (**Figure 10C**) induced by SNI were suppressed by the repeated administration of S1RA when measured on day 7 after surgery, 30 min after its administration.

The antineuropathic effects induced by repeated treatment with S1RA were still significant in all three sensory modalities explored (compared to treatment with the vehicle only) on day 10, 12 h after the last administration of S1RA (**Figure 10A–C**). However, the antineuropathic effects of S1RA disappeared in longer periods after treatment was discontinued: allodynia and hyperalgesia values on days 11 and 14 were indistinguishable from those in the vehicle-treated group (**Figure 10A–C**).

**Figure 11:**
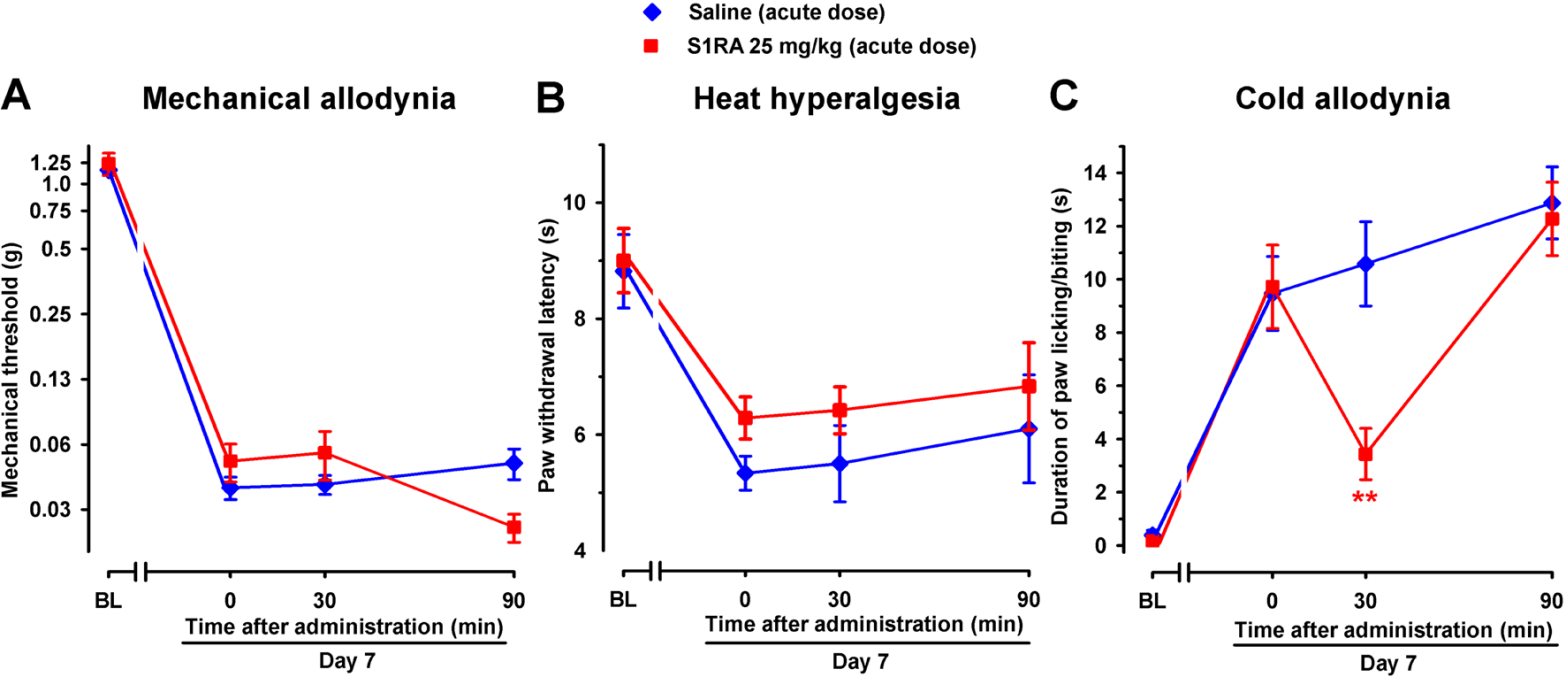
Time course of the effects of a single i.p. injection of S1RA (25 mg/kg) or saline on mechanical allodynia **(A)**, heat hyperalgesia **(B)**, and cold allodynia **(C)** in female WT mice with SNI 7 days after surgery. Responses were recorded in each animal before SNI (baseline, BL) and 7 days (day 7) after surgery. On day 7 the responses to test stimuli in the paw ipsilateral to the surgery were recorded immediately before (time 0) and at two times (30 and 90 min) after injection of the drug or saline. Each point and vertical line represent the mean ± SEM of the values obtained in six to eight animals. Statistically significant differences between S1RA- and saline-treated groups at the same time after treatment: **P < 0.01 (two-way repeated-measures ANOVA followed by Bonferroni test).

In contrast, acute treatment with S1RA (25 mg/kg, i.p.) had no significant effect on mechanical or heat hypersensitivities (**Figure 11A and B**), but significantly inhibited SNI-induced cold hypersensitivity (**Figure 11C**), in agreement with the previously commented higher sensitivity of S1RA effects on SNI-induced cold allodynia with respect to mechanical and heat hypersensitivity. The effects of this dose of S1RA lasted longer when administered s.c. than i.p. (compare **Figure 3C** and **11C**), suggesting a faster drug elimination of the later.

Taking into account that the acute administration of a dose of S1RA which lacks of effect on mechanical and heat hypersensitivity and induced only a transient effect on cold allodynia, but the repeated treatment with this same dose of S1RA induced a marked and long-lasting effect on the three outcomes examined, we conclude that repeated treatment with this drug results in an improvement of its effects.

### Concentration of S1RA in Plasma and Brain Tissue After Repeated Administration

**Figure 12:**
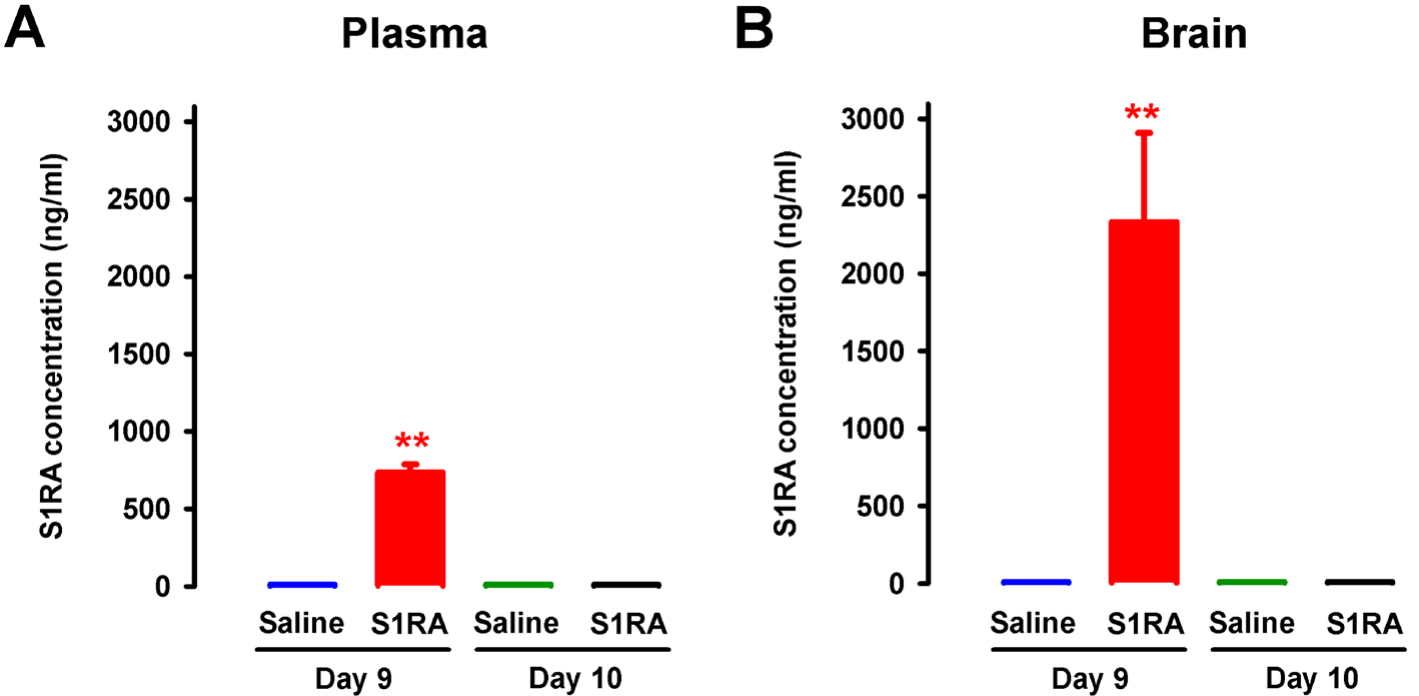
Concentrations of S1RA in plasma and brain tissue after its repeated administration in female WT. The levels of S1RA were measured by high-performance liquid chromatography–triple quadrupole mass spectrometry (HPLC-MS/MS) in plasma **(A)** and brain homogenates **(B)**. Mice were treated from day 0 to day 9 twice daily with either saline or S1RA (25 mg/kg, i.p.). Plasma and brain samples were obtained on day 9 (30 min after S1RA or saline administration) and day 10 (12 h after S1RA or saline administration). Each bar and vertical line represent the mean ± SEM of the values obtained in five to six animals **(A** and **B)**. Statistically significant differences between the levels 30 min after S1RA administration and the rest of the experimental groups: **P < 0.01 (two-way ANOVA followed by Bonferroni test).

To test whether the sustained antinociceptive effects induced by the repeated administration of S1RA 12 h after the discontinuation of drug treatment (i.e., on day 10) was due to the presence of any drug remaining in the organism, the concentrations of S1RA in plasma and brain tissue were measured 30 min and 12 h after the last dose of S1RA. On day 9 of repeated treatment, 30 min after the last administration of S1RA (25 mg/kg, i.p.), we found significant levels of this σ_1_ antagonist in both plasma and brain, with a much higher concentration in the latter (red bars in **Figure 12A and B**). In contrast, 12 h after the last administration, we observed no appreciable levels of this σ_1_ antagonist in any sample analyzed (**Figure 12A and B**).

## Discussion

The main findings of the present study are that: 1) pharmacological antagonism or genetic inactivation of σ_1_ receptors reduces neuropathic pain induced by peripheral nerve transection (SNI model); 2) the ameliorative effects on SNI-induced hypersensitivity to mechanical and heat stimuli (but not to cold stimuli) produced by σ_1_ receptor antagonism are mediated by modulation of the endogenous opioid system; and 3) repeated treatment with S1RA induces prolonged ameliorative effects which lasted longer than the presence of the drug in the organism.

Basal sensitivity to mechanical, cold, and heat stimulation in mice lacking σ_1_ receptors, in the absence of nerve injury, did not differ from that in WT mice. This is in agreement with previous studies (de la Puente et al., 2009; Nieto et al., 2012) and suggests that the basic mechanisms of nociceptive transduction are intact in mice lacking σ_1_ receptors. We showed that WT mice after SNI surgery developed mechanical, cold, and heat hypersensitivities with time courses similar to those previously reported in mice (Bourquin et al., 2006; Guida et al., 2015; Liu et al., 2015; Cobos et al., 2018). In addition, the extent of the sensory hypersensitivity was similar between female and male WT mice. In mice lacking σ_1_ receptors from either sex, however, SNI surgery induced a very different pattern of painful hypersensitivity: these mutant mice did not develop cold allodynia and showed significantly less mechanical allodynia, whereas they developed heat hyperalgesia normally. This is consistent with previous work in other neuropathic pain models, which found that σ_1_-KO mice had significantly reduced mechanical and cold allodynia induced by chemotherapy (Nieto et al., 2012) or by partial sciatic nerve ligation (de la Puente et al., 2009); the latter study also found that heat hyperalgesia developed normally (de la Puente et al., 2009). However, it was recently reported that σ_1_-KO mice showed, in addition to the reduction in mechanical allodynia, a significant attenuation of heat hypersensitivity induced by spinal cord injury (Castany et al., 2018) or diabetic neuropathy (Wang et al., 2018). Taken together, studies with σ_1_-KO mice suggest that the role of σ_1_ receptors during neuropathic pain depends on the sensory modality explored and the type of injury.

The acute pharmacological antagonism of σ_1_ receptor with S1RA administration, once the painful neuropathy was fully established in WT mice, significantly (although partially) attenuated mechanical allodynia and fully reversed cold hypersensitivity induced by SNI. In addition, and in contrast to our findings with σ_1_-KO mice, acute S1RA administration also abolished SNI-induced heat hyperalgesia in female or male WT mice. Previous studies in other neuropathic pain models reported that the systemic acute administration of σ_1_ antagonists (including S1RA) reversed not only neuropathic cold and mechanical allodynia (Nieto et al., 2012; Romero et al., 2012; Gris et al., 2016; Espinosa-Juárez et al., 2017b; Paniagua et al., 2017; Castany et al., 2018; Wang et al., 2018) but also heat hyperalgesia (Díaz et al., 2012; Romero et al., 2012; Paniagua et al., 2017; Castany et al., 2018; Wang et al., 2018), findings which are also in clear agreement with our results. Therefore, there is an apparent divergence between the reduction in heat hyperalgesia in S1RA-treated WT mice, and the normal development of heat hypersensitivity in σ_1_-KO mice after SNI. This inconsistency cannot be explained by a nonspecific off-target effect of S1RA, because here we show that the acute ameliorative effects induced by S1RA in all three sensory modalities explored in the present study were abolished by the selective σ_1_ agonist PRE-084, which did not show any effects “per se,” as previously described in a different neuropathic pain model (Espinosa-Juárez et al., 2017b). In addition, the administration of S1RA to σ_1_-KO mice had no effect on SNI-induced mechanical and heat hypersensitivity. Therefore, these results argue in favor of a σ_1_-mediated action induced by this drug. In fact, it is known that S1RA has exquisite selectivity for σ_1_ receptors (Romero et al., 2012). The discrepancy between the effect of genetic and pharmacological inhibition of σ_1_ receptors on heat hypersensitivity was also found in other studies of neuropathic pain (de la Puente et al., 2009; Romero et al., 2012) and during inflammatory pain (Tejada et al., 2014). In addition, conflicting results between σ_1_ knockout and pharmacological antagonism have been reported in the modulation of opioid-induced analgesia in nociceptive heat pain (Vidal-Torres et al., 2013). Studies of pain mechanisms that used the genetic and pharmacological inhibition of other receptors have also obtained contradictory results (Petrus et al., 2007; Bonin et al., 2011), which have been attributed to compensatory mechanisms developed by mutant mice. Therefore, this issue appears to be a general concern in experiments with knockout animals. Taking into account these antecedents, we suggest that σ_1_-KO mice develop these purported compensatory mechanisms in heat pain pathways but not in other pain pathways in which the knockout replicated the effects of σ_1_ antagonists.

After peripheral nerve injury, neuroinflammatory processes occur with the recruitment of myriad immune cells at the site of injury (Treutlein et al., 2018) and in the dorsal root ganglia (Kwon et al., 2013), where the somas of peripheral sensory neurons are located. These immune cells release a variety of inflammatory mediators that contribute to neuropathic pain, but they also produce EOPs which have analgesic potential (reviewed in Refs. Ji et al., 2014; Stein, 2016; Tejada et al., 2018). In our experimental conditions, opioid antagonism caused by the administration of naloxone or its peripherally restricted analog naloxone methiodide did not exacerbate pain hypersensitivity in any sensory modality explored, suggesting that the tonic endogenous activity of the opioid system is limited in SNI mice. Importantly, the ameliorative effects induced by S1RA in SNI-induced mechanical and heat hypersensitivity were reversed by both naloxone and naloxone methiodide. These results suggest that σ_1_ inhibition ameliorates SNI-induced mechanical and heat hypersensitivity through a mechanism dependent on peripherally produced EOPs, whose actions are tonically limited by σ_1_ receptors. This dependence on the peripheral opioid system of the effects induced by S1RA on mechanical and heat hypersensitivity was seen in both female and male mice, indicating a lack of sexual dimorphism in these effects. It was recently reported that σ_1_ antagonism produced opioid-dependent antihyperalgesic effects during inflammation by enhancing the action of EOPs released by immune cells that accumulate at the inflamed site (Tejada et al., 2017). Interestingly, σ_1_ receptors are expressed in the somas of all peripheral sensory neurons (Mavlyutov et al., 2016; Montilla-García et al., 2018), at a much higher density than in central pain-related areas (Sánchez-Fernández et al., 2014). In light of these antecedents, it can be hypothesized that our results for the effects of S1RA on neuropathic mechanical and heat hypersensitivity may also be attributable to the enhancement, by σ_1_ antagonism, of the peripheral antinociceptive actions of EOPs produced by immune cells. Further research is guaranteed to determine the exact EOP/EOPs involved in the opioid-dependent effects induced by σ_1_ antagonism during neuropathic pain.

It has been reported that peripheral immune cells do not contribute equally to every modality of sensory hypersensitivity after peripheral nerve injury. In fact, peripheral macrophages and T-cells promote both mechanical allodynia and heat hyperalgesia (Kobayashi et al., 2015; Cobos et al., 2018), whereas their influence in cold allodynia is very limited, which suggest that it is due to neuronal mechanisms rather than to neuroimmune interactions (Cobos et al., 2018). Here we show that the effects of S1RA on cold allodynia in either female or male mice are independent of opioid activation, which is consistent with the known inhibitory effects of S1RA on neuronal sensitization (Romero et al., 2012; Paniagua et al., 2017). In this connection it was previously reported that the effects of σ_1_ antagonists in other pain models such as capsaicin-induced secondary hypersensitivity (Entrena et al., 2009) or formalin-induced pain (Tejada et al., 2017) were not sensitive to naloxone treatment. Taking into account the wide variety of protein partners (other than opioid receptors) that benefit from the chaperoning actions of σ_1_ receptors (reviewed in Refs. Su et al., 2016; Sánchez-Fernández et al., 2017), it is not surprising that multiple opioid and nonopioid mechanisms simultaneously participate on the ameliorative effects of σ_1_ antagonism.

We also found that whereas morphine only partially reversed mechanical allodynia, it was able to fully suppress heat and cold hypersensitivity induced by SNI—effects which resemble those induced by S1RA. The effects of morphine on mechanical and heat hypersensitivity, but not on cold allodynia, were sensitive to the peripheral opioid antagonist naloxone methiodide. These results suggest that peripheral opioid effects are markedly weaker in cold allodynia than in mechanical or heat hypersensitivity, and are consistent with the absence of peripherally mediated opioid effects on the inhibition of cold allodynia induced by S1RA.

We also tested the effects of the repeated administration of S1RA, which was administered preemptively before surgery and subsequently administered twice a day during the next 9 days. Plasma levels of S1RA in mice after repeated treatment with this drug were similar to or lower than the levels of this drug in humans after daily oral S1RA treatment at therapeutic doses (Abadias et al., 2013; Bruna et al., 2018). We found that the sustained administration of S1RA induced prolonged ameliorative effects on the hypersensitivity to mechanical, heat, and cold stimuli, without any evidence of tolerance to the antineuropathic effects during the evaluation period. This may be relevant given that some effects of S1RA, as shown in the present study, involve the opioid system, and it is well known that sustained opioid treatment induced analgesic tolerance (Morgan and Christie, 2011). Therefore, although we show that the effects of σ_1_ antagonism are partially mediated by the opioid system, this does not necessarily imply the development of analgesic tolerance.

We evaluated the effects of the repeated administration of S1RA starting before neuropathic pain was established, and found that it had marked effects on mechanical, heat, and cold hypersensitivity. However, when the same dose of S1RA was acutely administered once neuropathic pain was fully established, its effects were limited and observed only in cold allodynia. Therefore, S1RA showed higher efficacy after repeated treatment than after a single treatment. It is unlikely that the greater effects induced by repeated treatment of S1RA were due to drug accumulation, since we previously showed that repeated treatment with the same protocol as in the present study did not result in increased levels of S1RA with time (Romero et al., 2012). In addition, here we show that 12 h after treatment was discontinued, there were no appreciable levels of S1RA in either plasma or brain tissue, indicating the complete elimination of this compound between doses. Interestingly, although no S1RA remained in the organism 12 h after its last administration, drug effects were still significantly evident in all three sensory modalities. Our results are consistent with previous studies in which the repeated administration of σ_1_ antagonists (including S1RA) consistently induced a long-lasting reduction of the development of mechanical, cold, and heat hypersensitivity in models of neuropathic pain of different etiologies (Nieto et al., 2012; Gris et al., 2016; Paniagua et al., 2017). It is unclear whether these prolonged effects induced by the repeated treatment with S1RA might be due to the production of an active metabolite not detected in our determinations. However, it is known that σ_1_ receptors can influence gene transcription, which might account for the long-lasting effects observed (Tsai et al., 2015). In fact, repeated treatment with the σ_1_ agonist PRE-084, which is chemically unrelated to S1RA, had long-lasting proallodynic effects (Entrena et al., 2016). Taken together, these results suggest that the repeated treatment with σ_1_ ligands might have sustained pharmacodynamic effects, not restricted to S1RA and its possible active metabolites, although further experiments are needed to clarify this issue. Regardless of the precise mechanism, our data suggest that repeated treatment with S1RA may have potential therapeutic utility in the context of neuropathies induced by nerve transection during surgery, when the precise time of nerve injury can be anticipated and preventive treatment can be given.

In summary, this study demonstrates that σ_1_ antagonism may be a potentially effective therapeutic tool to inhibit neuropathic pain induced by peripheral nerve transection. In addition, our findings support the notion that σ_1_ antagonism induces both opioid-dependent and -independent effects during neuropathic pain.

## Author Contributions

EJC and FRN designed research; IB-C, GP, SY, DC, and FRN performed research; IB-C, SY, FRN, and JMB analyzed data; IB-C, JMB, EJC, and FRN wrote the paper. All authors read and approved the final version of the manuscript.

## Funding

IB-C was supported by an FPU grant from the Spanish Ministry of Education, Culture, and Sports. This study was partially supported by the Spanish Ministry of Economy and Competitiveness (MINECO, grant SAF2016-80540-R), the Junta de Andalucía (grant CTS 109), and funding from Esteve and the European Regional Development Fund (ERDF). This research was done in partial fulfillment of the requirements for the doctoral thesis of IB-C.

## Conflict of Interest Statement

SY was employed by Esteve.

The remaining authors declare that the research was conducted in the absence of any commercial or financial relationships that could be construed as a potential conflict of interest.

The authors declare that this study received funding from Esteve. This company had no role in study design, data collection and analysis, decision to publish, or preparation of the manuscript.
